# Effects of Bisphenols on Testicular Steroidogenesis

**DOI:** 10.3389/fendo.2020.00373

**Published:** 2020-06-30

**Authors:** Federica Barbagallo, Rosita A. Condorelli, Laura M. Mongioì, Rossella Cannarella, Antonio Aversa, Aldo E. Calogero, Sandro La Vignera

**Affiliations:** ^1^Department of Clinical and Experimental Medicine, University of Catania, Catania, Italy; ^2^Department of Experimental and Clinical Medicine, “Magna Graecia” University, Catanzaro, Italy

**Keywords:** bisphenols, BPA, endocrine disruptors, testicular steroidogenesis, spermatogenesis

## Abstract

Over the last decades, the adverse effects of human exposure to the so-called “endocrine disruptors” have been a matter of scientific debate and public attention. Bisphenols are synthetic chemicals, widely used in the manufacture of hard plastic products. Bisphenol A (BPA) is one of the best-known environmental toxicants proven to alter the reproductive function in men and to cause other health problems. Consumer concern resulted in “BPA free” products and in the development of bisphenol analogs (BPA-A) to replace BPA in many applications. However, these other bisphenol derivatives seem to have effects similar to those of BPA. Although a number of reviews have summarized the effects of BPA on human reproduction, the purpose of this article is to review the effects of bisphenols on testicular steroidogenesis and to explore their mechanisms of action. Testicular steroidogenesis is a fine-regulated process, and its main product, testosterone (T), has a crucial role in fetal development and maturation and in adulthood for the maintenance of secondary sexual function and spermatogenesis. Contradictory outcomes of both human and animal studies on the effects of BPA on steroid hormone levels may be related to various factors that include study design, dosage of BPA used in *in vitro* studies, timing and route of exposure, and other confounding factors. We described the main possible molecular target of bisphenols on this complex pathway. We report that Leydig cells (LCs), the steroidogenic testicular component, are highly sensitive to BPA and several mechanisms concur to the functional impairment of these cells.

## Introduction

Over the last decades, the adverse effects of human exposure to the so-called “endocrine disruptors” have been a matter of deep debate by the scientific community and the layman. Particular attention has been paid to their toxicity on the reproductive function. Bisphenol A [2,2-bis(4-hydroxyphenyl)propane] (BPA) is among the most well-known endocrine disruptors proven capable of impairing the male reproductive function and to cause other health problems. BPA is an organic synthetic compound, including the group of dyphenylmenthane derivatives and bisphenols, widely used in the manufacture of hard plastic products. BPA has been used since the 1950s, in food packaging, industrial materials, dental sealants, personal hygiene products, and thermal receipts (1, 2). A significant exposure to BPA for children is given by toys, books, and feeding bottles (3, 4). BPA penetrates the body through the skin, inhalation, and the digestive system (5). Once adsorbed, BPA is then metabolized by the liver and excreted with the urine in 24 h (2). Despite the rapid metabolism, BPA can accumulate in different tissues (6). Consumer concern for BPA effects on health resulted in “BPA free” products and in the development of bisphenol analogs to replace BPA in many applications. However, these compounds seem to have endocrine disrupting capabilities similar to BPA and their impact on reproduction has been little investigated (7–9).

BPA seems to influence fetal testis development and predispose to the testicular dysgenesis syndrome (TDS). This syndrome may manifest itself not only at birth with cryptorchidism and hypospadias, but also in adulthood when it shows up with testicular tumors, hypogonadism, and/or infertility (10). Current evidence suggests that BPA can cause testicular histological abnormalities, which encompass dysregulated proliferation and apoptosis of Leydig cells (LCs) and alteration of steroidogenesis (11). In mice, pubertal exposure to high doses of BPA causes LC and germ cells apoptosis, resulting in underdeveloped testis with histopathological changes including atrophied seminiferous tubules, decreased number of late spermatids, and increased karyopyknotic cells (12). The reduction of testicular weight and the alteration of spermatogenesis persist till adulthood, long after the period of BPA exposure (12). The gestational period is a sensitive window of exposure to BPA. Male rats maternally exposed to BPA from gestation to the postnatal period have low testicular weight and testosterone (T) levels in the testicular interstitial fluid in adulthood (13). These effects may involve different molecular pathways discussed in section Bisphenol A Molecular Mechanisms of Action on Testicular Steroidogenesis.

Many studies have investigated the effects of BPA on human reproduction and extensive reviews have addressed the strength of the evidence on BPA toxicity (9, 10, 14, 15). Contradictory outcomes may depend on several factors including study design, BPA dose, timing, and route of exposure and other confounding factors (15). Several mechanisms of action have been described. First of all, BPA exhibits weak estrogenic and antiandrogenic proprieties. It binds to both estrogen receptors (ERs), ERα and ERβ (1, 10), and at high concentrations, BPA binds to the androgen receptor (AR) on which it acts as an antagonist (16). In addition to binding to the ARs, it disturbs the hypothalamic–pituitary–testicular axis and modulates gene expression and the enzymatic activity of testicular steroidogenesis (16). Furthermore, exposure to BPA is also associated with a decrease in the activity of the antioxidant system, resulting in increased oxidative stress, the most common cause of sperm damage (17, 18). Although several studies have supported the harmful effects of BPA on testicular function, its mechanism remains not fully understood.

The purpose of this article is to review the evidence on the relationship between bisphenols and testicular steroidogenesis, focusing on their mechanism(s) of action on LCs function.

## Testicular Steroidogenesis

The testis is a complex endocrine organ regulated by intra- and extra-testicular pathways that interact synergistically (19). LCs have a crucial role in the regulation of steroidogenesis and spermatogenesis. LCs produce testosterone (T), which has a main role in fetal development and maturation. During the masculinization programming window, the fetal testes begin to produce T, which allows male gonadal differentiation and development (20). Hence, T is necessary for the maintenance of secondary sexual function and spermatogenesis (21). Intratesticular T levels are approximately 100 times higher than the levels found in systemic circulation (22). The high local production rate of T implies the need for its intratesticular transport from LCs to Sertoli cells which nourish and support the development of the germinal cells during the various stages of spermatogenesis (23). LCs derive from mesenchymal cells located in the interstitial compartment of the testis. Their development occurs through three different stages during which they are called progenitor, immature, and adult LCs. Apoptosis seems to have a main role in maintaining a constant population of LCs, although other mechanisms may be involved (9).

**Figure 1:**
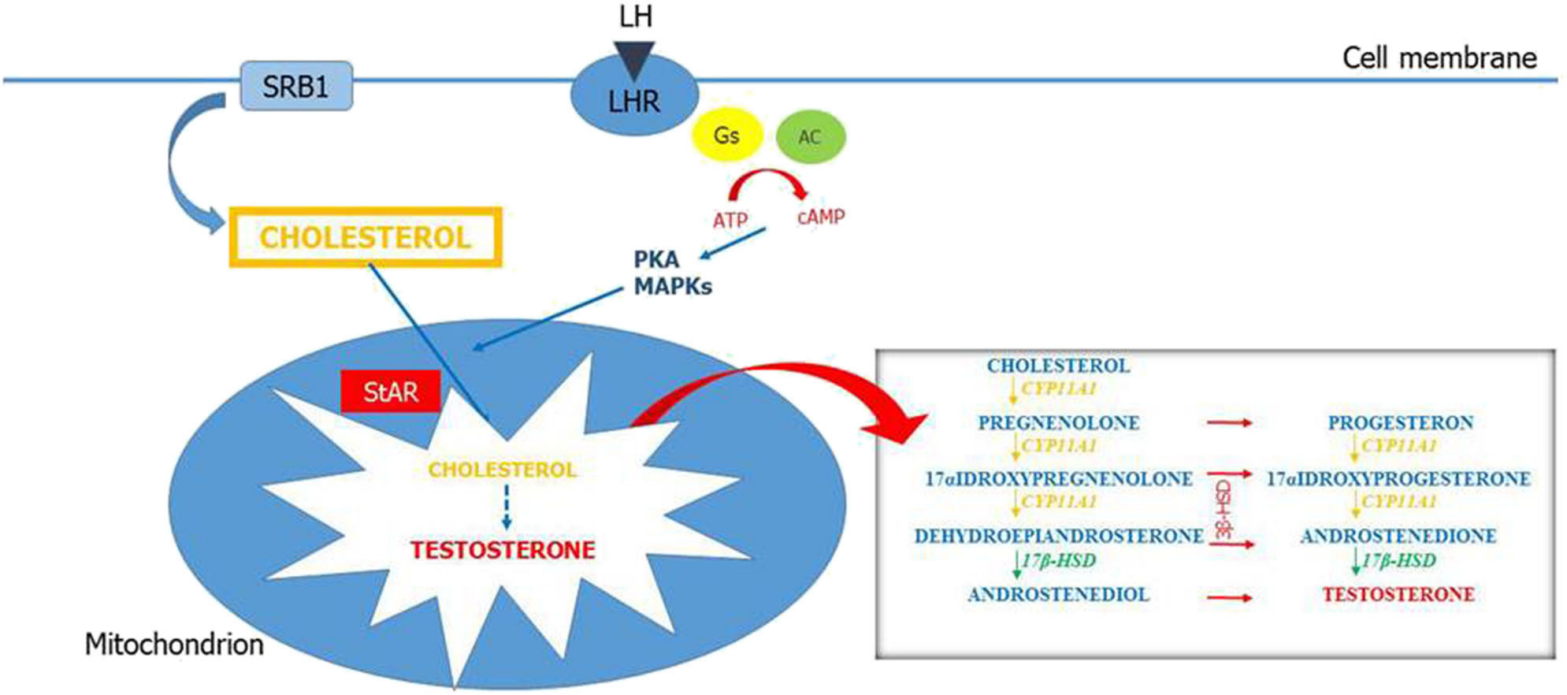
Leydig cell steroidogenesis. LH binds to its receptors (LHR) on the Leydig cell (LC) membrane. This results in activation of Gs protein and adenylyl cyclase and increased concentration of intracellular cAMP. cAMP stimulates the mobilization and transport of cholesterol within the mitochondria in part by activating PKA and MAPK signaling. The first source of cholesterol for steroidogenesis is via uptake of cholesteryl esters from high-density lipoprotein (HDL) by the scavenger receptor SR-B1. Steroidogenic acute regulatory enzymes (StARs) regulate cholesterol transport from the outer to the inner mitochondrial membrane. At the inner mitochondrial membrane, cholesterol is converted into pregnenolone by CYP11A1 and pregnenolone is converted into testosterone by enzymes in the smooth endoplasmic reticulum (3β-HSD, CYP17A1, and 17β-HSD).

LCs produce T in response to the luteinizing hormone (LH). LH binding to the LH receptors (LHR) on LCs activates Gs protein and adenylyl cyclase, increasing cAMP levels. cAMP acts as a key second messenger and upregulates the expression of genes related to the steroidogenesis (24). The steroidogenesis consists in a complex multi-enzyme process by which precursor cholesterol is converted to biologically active steroid hormones in a tissue-specific manner (Figure 1). Cholesterol can be synthesized in the endoplasmic reticulum but the first source of this precursor for steroidogenesis is via uptake of cholesteryl esters from high-density lipoprotein by the scavenger receptor SR-B1 (25). Therefore, SR-B1 has a key role for the maintenance of cholesterol balance. The first step in steroidogenesis takes place within mitochondria. The steroidogenic acute regulatory protein (StAR) mediates the transport of cholesterol from the outer to the inner mitochondrial membrane (26). The StAR-mediated transport of cholesterol is a crucial step for steroidogenesis (27, 28) and appropriate concentrations of cAMP are necessary for the regulation of StAR expression (29). However, cAMP/PKA is not the only pathway that regulates StAR expression. Other factors such as steroidogenic factor, activator protein, and cAMP-response element-binding protein are also associated with StAR regulation (30). Then, cholesterol is metabolized to pregnenolone into the smooth endoplasmic reticulum through a cascade of reactions that are catalyzed by the cytochrome P-450 proteins. Pregnenolone is then converted to T by 3β-hydroxysteroid dehydrogenase (3β-HSD), 17α-hydroxylase/17,20 lyase (CYP17A1), and 17β-hydroxysteroid dehydrogenase (17β-HSD). This complex process of steroidogenesis itself can be responsible for the increase of reactive oxygen species (ROS) (31). Thus, the normal products of steroidogenesis can act as pseudosubstrates and interact with P-450 enzymes, resulting in a pseudosubstrate–P-450–O_2_ complex, which is a source of dangerous free radicals (32).

## Bisphenols and Testicular Steroidogenesis

### Effects of BPA on Steroid Hormone Levels

Experimental studies in male animals have shown that exposure to BPA is associated with altered hormone levels suggesting direct effects of BPA on LCs. However, these data are discordant. Low-dose BPA decreased T levels in CD-1 mice exposed during perinatal and postnatal periods (33), but not in adult C57BL/6 mice exposed *in utero* (34). In addition, low-dose BPA lowered T levels in Holtzman rats exposed during gestation or in the neonatal age (35, 36) and albino (37) and Wistar (38) rats exposed in adulthood. In contrast, by examining the gestational and neonatal exposure of low-dose BPA in Long–Evans (39) or Sprague–Dawley (SD) rats (40, 41), the levels of T did not change. Treatment with increasing concentrations of BPA (1 to 1,000 nM) did not significantly lower basal or hCG-stimulated T secretion by primary culture of LCs of young adult male rats (42). However, although Sánchez et al. reported that low-dose BPA did not decrease T levels in Wistar rats, dihydrotestosterone levels decreased (43). Gamez et al. reported that exposure to low-dose BPA led to an increase in serum LH and FSH levels in young Wistar rats (44). Nevertheless, another study in adult Wistar rats showed that exposure to BPA decreased serum T, LH, and FSH levels, but increased the levels of 17β-estradiol (E_2_) (45). In two studies in SD rats, postnatal exposure to low-dose BPA decreased serum T and E_2_ levels (46). BPA exposure lowered T levels in Swiss albino and C57BL/6 mice, but at variable dosage between 0.5 μg/kg and 100 mg/kg (47, 48). Sadowski et al. described a decrease in FSH concentrations in Long–Evans rats at weaning, after exposure to BPA at both 4 and 400 μg/kg/day (49). An *in vitro* study conducted on fetal testes explanted from mice, rats, and humans demonstrated that exposure to 10 nM of BPA was enough to decrease basal T secretion in human fetal testes, but higher concentrations were required in rats and mice (10 and 1 μM, respectively) (50).

The epidemiological studies evaluating the effects of BPA exposure on serum hormone levels in men have also shown conflicting results. In the INChianti adult population study, Galloway et al. found a correlation between higher urinary BPA concentrations and higher serum T, but not E_2_ levels in 307 Italian men living in Chianti, Italy (51). Another study, conducted on 308 young men from Denmark's general population, reported that higher urinary BPA concentration was associated with a significant increase of LH, T, and E_2_ levels (52). In contrast, in a cross-sectional study of 290 men, Zhou et al. found that increased serum BPA concentrations were statistically significantly associated with the reduction of androstenedione, free T and free androgen index (FAI) levels, and with the increase of sex hormone-binding globulin (SHBG) levels (53). Two cross-sectional studies, respectively, of 167 and 302 men, did not report any associations between BPA and T concentrations (54, 55). According to Meeker and colleagues, men with elevated urinary BPA concentrations had higher FSH and lower inhibin B levels with a higher FSH/inhibin B ratio and a lower E_2_/T ratio (54). Mendiola et al. found that higher urinary BPA levels were associated with lower FAI and FAI/LH and free T/LH ratios in fertile men (55). Two cross-sectional studies reported that urinary BPA levels were associated with higher SHBG in men occupationally exposed to BPA (56, 57). The NHANES 2011-2012 study showed an inverse correlation between urinary BPA levels and serum T concentrations in male adolescents (58). However, a retrospective cohort study did not find any effects on hormone levels in boys aged 8 to 14 years after prenatal or childhood exposure to BPA (59).

Although these results are controversial, they suggest that BPA alters steroid hormones pathways in men.

### BPA Molecular Mechanisms of Action on Testicular Steroidogenesis

Although both animal and human studies support the harmful effects of BPA on steroid hormones, the mechanism of action of BPA in negatively interfering with testicular steroidogenesis remains unclear. Since LCs are the site of testicular steroidogenesis, several studies have been conducted on these cells to investigate the effects of BPA. In Wistar/ST pubertal rats, continuous exposure to BPA at high doses reduced the number of LCs and the expression of steroidogenic enzymes in these cells (60). In contrast, Long–Evans rats exposed to a low dose of BPA during gestation and at birth had an increase in the number of LCs in adulthood through the upregulation of mitogen factors. However, although a low dose of BPA increased LC proliferation, the expression of steroidogenic enzymes and T biosynthesis decreased (61). Chen et al. reported that BPA did not stimulate staminal LC proliferation, but it induced the differentiation of stem LCs into more mature LCs. They used an *in vivo* ethane dimethane sulfonate (EDS)-induced LC regeneration model to mimic the pubertal development of LCs. They treated rats with EDS to eliminate LCs and then they injected BPA within the testis. The intratesticular injection of BPA avoided possible interference of hypothalamus and pituitary. The results of this study showed that BPA significantly increased the number of 11β-HSD1-positive cells, which is a biomarker for LCs at an advanced stage. Thus, BPA promoted the differentiation of staminal LCs, increasing T production and upregulating LC-specific genes (LHCGR, StAR, CYP11A1, HSD3B1, CYP17A1, HSD17B3, and HSD11B1). These findings suggest a possible role of BPA in sexual precocious puberty in males (62). Exposure to high doses of BPA (480 and 960 mg/kg/day at postnatal days 31–44) has been reported to induce apoptosis in Leydig and germ cells *via* the upregulation of Fas, FasL, and caspase-3 (12). The apoptosis of LCs was associated with a decreased testicular testis weight and histopathological changes, which persisted into adulthood (12). In another study, Thuillier et al. reported that SD rats exposed *in utero* to BPA had an increase number of LCs but did not present significant change in serum T levels (63). Moreover, BPA can also induce *Nur77* gene expression, an orphan nuclear receptor that plays an important role in the regulation of LH-mediated steroidogenesis, altering LC steroidogenesis (64). BPA induced Nur77 gene expression via PKA and MAPK signaling pathways in a time- and dose-dependent manner. BPA-mediated Nur77 expression resulted in the upregulation of steroidogenesis both *in vitro* and *in vivo*, with a significant increase of T synthesis (two-fold) (64).

The inhibition of testicular steroidogenesis by BPA can also be associated with a decreased LH secretion. Akingbemi et al. reported that Long–Evans rats exposed to low doses of BPA (2.4 μg/kg/day) from postnatal days 21–35, decreased both serum LH and T levels, downregulating pituitary LHβ expression but increasing ERβ pituitary mRNA levels (13).

The expression of LH and FSH receptors may also be altered by BPA. Li et al. showed that treatment of adult male zebrafish (Danio rerio) by 500 ng/L BPA for 7 weeks downregulated the expressions of FSHr and LHCGr (65). For the first time, Roelofs et al. demonstrated that BPA, BPF, and TBBPA showed clear glucocorticoid receptor antagonism, other than AR antagonism. They also found that bisphenol analogs upregulated the 5αRed1 gene expression, suggesting a redirection of steroidogenesis, which may have significant consequences for fetal testis development and function (7).

Within the steroid hormone biosynthetic pathway, steroidogenic enzymes are recognized as important targets for the actions of endocrine-disrupting chemicals. Several studies showed that BPA decreases the expression of steroidogenic enzymes (33, 41, 60, 61, 66, 67). Moreover, some compounds, including BPA, seem to disturb steroidogenesis by inhibiting the cAMP pathway. Nikula et al. analyzed the effects of BPA at micromolar concentration in cultured mouse Leydig tumor cells (mLTC-1). BPA did not have any effects on hCG binding to LH receptors, but it inhibited LH-receptor-mediated signal transduction by decreasing hCG-stimulated cAMP. Specifically, they found that after preincubation of mLTC-1 cells for 48 h with different doses of BPA, hCG-stimulated cAMP and progesterone production was inhibited. Whereas, preincubation with 17β-estradiol inhibited progesterone production but had no effect on cAMP. Thus, the effects of BPA did not seem to be estrogen-related (68). Moreover, the inhibitory effect of BPA could not be seen when cAMP formation was directly stimulated by forskolin (Fk) or through Gs protein by cholera toxin (CT), and when steroidogenesis was directly activated by 8-Br-cAMP, which can penetrate the plasma membranes and directly activate the protein kinase A. These results suggested that the negative effect of BPA is exerted between the LH receptor and the adenylate cyclase. Accordingly, Feng et al. found that BPA exposure inhibited progesterone secretion in hCG-stimulated mouse Leydig tumor cell line (mLTC-1) by decreasing SR-B1 and P450scc expression due to the adverse effects on cAMP. Moreover, lower SR-B1 levels cause a reduction in cholesterol levels within LCs that alters steroidogenesis (69). The role of StAR is instead controversial. According to Feng et al. (69), StAR seems not be the molecular target of BPA. Similarly, male rats exposed to BPA showed decreased T levels but did not exhibit significant changes in StAR expression (61). However, other previous studies have reported that BPA decreased StAR expression in cell culture *in vitro* (15, 33, 47), but, in contrast, other studies have shown that StAR expression is upregulated (41, 65). Takamiya et al. reported that StAR gene expression increased in the presence of both hCG (10 μg/L) plus BPA (10^−5^ M) or by hCG alone, but was not influenced by BPA alone. They found that BPA had only a weak modulating effect on gene expression of hCG-stimulated mLTC-1 cells (70). Li et al. showed that the exposure of adult male zebrafish to low doses (0.22–2.2 nM) of BPA for 7 weeks resulted in abnormal expression of genes involved in testicular steroidogenesis, specifically of 3β-HSD1, CYP17A1, and CYP11C1 (65). Samova et al. found that BPA significantly and dose-dependently affected the functions of 3β-HSD and 17β-HSD in the testis of inbred Swiss strain male albino mice (67). Ye et al. reported that BPA significantly inhibited 3β-HSD, CYP17A1, and 17β-HSD3 activities in both human and rat testis. However, the inhibition of 17β-HSD3 activity was much weaker compared with that on the other two enzymes. They also found that human enzymes were more sensitive to BPA (71). Specifically, their results suggested that BPA did not exert a competitive inhibition of 3β-HSD against its substrate (pregnenolone), but it competed with the cofactor NAD+ in the cofactor binding site of the enzyme, whereas BPA inhibition of CYP17A1 was mixed type for enzyme substrate progesterone, indicating a combination of two different types of reversible enzyme inhibition, both competitive and uncompetitive (71). Additionally, not only BPA, but also bisphenol S (BPS) and bisphenol F (BPF) exposure decreased T production in fetal mouse testis by inhibiting mRNA expression of StAR, 3β-HSD, and cytochrome P45017A1 (CYP17A1), but not of P450scc (72). Moreover, Dankers et al. suggested that the changes in T secretion after BPA or TBBPA exposure were only partly due to alterations of steroidogenic enzyme expression. These authors hypothesized that the inhibition of ATP-binding cassette (ABC) transporters, expressed in the blood–testis barrier (BTB), may play a role in this process. The BTB divides the seminiferous epithelium into a basal and an apical compartment and provides structural and protective support for the differentiation of spermatogonia into spermatocytes. It consists of tight junctions, testis-specific atypical adherent junctions, desmosomes, and gap junctions. In the active part of BTB, ABC transporters are present to allow the passage of endogenous molecules involved in cellular signaling and to block the passage of dangerous compounds within the testes and to protect germ cells. The cellular membranes of LCs, Sertoli cells, and capillary endothelial cells are provided of these transporters. For this reason, the association between endocrine disruptors and ABC transporters has a strong toxicological impact (23). The breast cancer resistance protein (BCRP/*ABCG2*), the P-glycoprotein (P-gp/*ABCB1*), and the multidrug resistance proteins 1 and 4 (MRP1, 4/*ABCC1,4*) are the major efflux transporters in the BTB with a differential expression in the various parts of the BTB (23). LCs express P-gp, MRP1, and MRP4, but not BCRP in adult human testis (73, 74). Dankers et al. investigated the effects of BPA and of TBBPA (tetrabromobisphenol A) on BCRP, MRP1, MRP4, and P-gp. They found that TBBPA inhibited all these transporters; thus, it is considered a non-competitive transporter inhibitor, whereas BPA inhibited only BCRP activity. They also showed that BPA, but not TBBPA, is transported by BCRP (23). Interestingly, they found that, although exposure to BPA and TBBPA significantly increased T level in MA-10 cells, the effects on steroidogenic genes were not so significant. Thus, these authors hypothesized that the changes in T levels upon BPA or TBBPA exposure were associated with the inhibition of efflux of T precursors. Increased availability of these precursors, such as androstenedione or DHEA, could be responsible for the increased T levels found.

Moreover, many compounds increase the levels of ROS in the testis, altering steroidogenesis. Oxidative stress has also been found to induce apoptosis in LCs and germ cells (64). Recent studies have reported an inverse relationship between NOS activity and StAR expression (47). Chouhan et al. exposed Swiss albino mice to BPA at concentrations of 0.5, 50, and 100 μg/kg body weight/day intraperitoneally for 60 days. They showed that BPA upregulated the expression of iNOS, downregulating the expression of StAR in mouse testis (47). It was also supposed that BPA impaired steroidogenesis by decreasing testicular glucose levels (38). Glucose homeostasis is crucial for testicular spermatogenesis and steroidogenesis. D'Cruz et al. reported that low-dose BPA exposure impaired insulin signaling interacting with GLUT-2 and GLUT-8 and inhibiting the uptake in the testis (38).

Recently, a number of studies suggest epigenetic effects of BPA, including DNA methylation, histone modifications, and non-coding RNAs. Epigenetic mechanisms can have long-term effects and may be transmitted across several generations (75). Specifically, Gao et al. (76) have recently investigated the epigenetic effects of BPA on the expression of non-coding RNAs (e.g., microRNAs) in the regulation of testicular steroidogenesis. They used both cell culture and *in vivo* mouse models and showed that miR-146a-5p was expressed only in LCs, and this expression was significantly induced by BPA. Consequently, the high miR-146a-5p expression intensifies the negative effects of BPA on testicular steroidogenesis by directly targeting the 3′ UTR of Mta3 gene (76). Mta3 is a subunit of the Mi-2/nucleosome remodeling and deacetylase (NuRD) protein complex that is exclusively expressed in LCs (77). Specifically, Mta3 role in the control of testicular steroidogenic function is proven by its negative regulation by the high levels of circulated insulin (77). He et al. showed that a deficiency of Mta3 in LCs of diabetic mice was associated with low serum T level, indicating that Mta3 expression in LCs may be associated with androgen deficiency (77). Thus, the downregulation of mir-146a-5p/Mta3 cascade seems to be involved in steroidogenic alterations caused by BPA (76).

DNA methylation is one of the best characterized epigenetic mechanisms. Liu et al. investigated the effects of BPA on DNA methylation in rare minnow *Gobiocypris rarus*. DNA hypermethylation consists of an addition of a methyl group to the cytosine bases of DNA to form 5-methylcytosine and it may be associated with changes in gene expression. In their study, Liu et al. found that the global DNA methylation level was significantly increased in testis of male *G. rarus* exposed to BPA for 7 days. Then, they specifically analyzed the change in DNA methylation in the 5′ flanking region of the cytochrome P450 aromatase (CYP19A1A) gene. After 35-day exposure, the DNA methylation levels of CYP19A1A did not have significant change in the testis, whereas they significantly increased in the ovary (78).

## Conclusions

**Figure 2:**
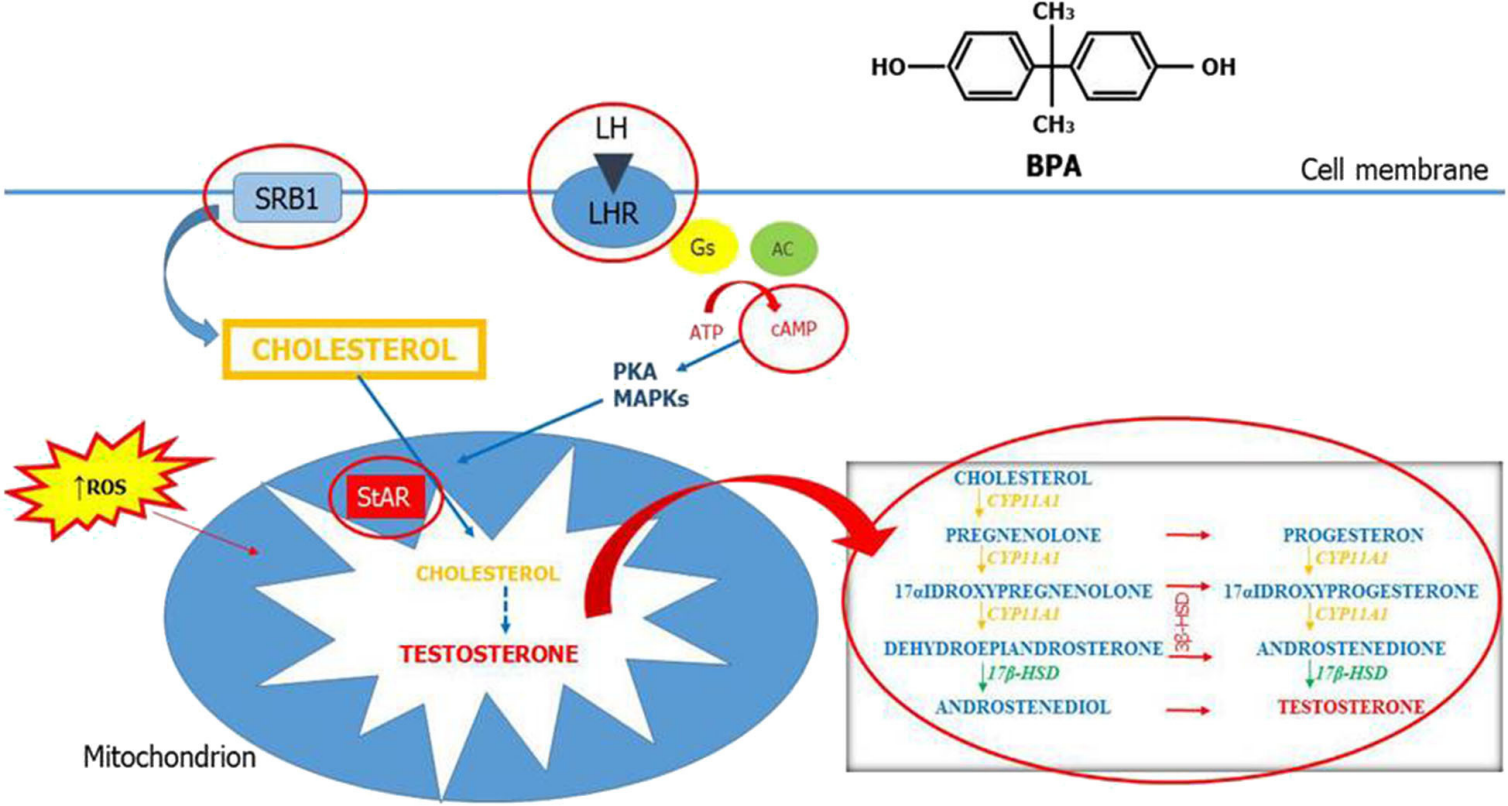
Mechanisms of action of bisphenol A on testicular steroidogenesis. Testicular steroidogenesis is a complex and fine-regulated process that bisphenol A (BPA) can perturb by acting with several mechanisms represented in this figure (circled in red).

This review summarizes the current evidences on the association between BPA and testicular steroidogenesis. Altogether, these results show that LCs are very sensitive to BPA and that several mechanisms concur to the functional impairment of these cells. Testicular steroidogenesis is a complex and fine-regulated process and each component of this pathway may be the molecular target of BPA. The main possible sites of BPA action are summarized in Figure 2. The conflicting results of both human and animal studies may be related to various factors that include study design, dose of BPA, timing and route of exposure, and other confounding factors. This review confirms that the widespread use of bisphenols is certainly dangerous for testicular development and function and that a reduction of its use is necessary to preserve male sexual and reproductive health.

## Author Contributions

FB, SL, and AC: concept and design. RAC, LM, and RC: articles research. FB: writing of the original draft. SL, AC, and AA: final approval. All authors contributed to the article and approved the submitted version.

## Conflict of Interest

The authors declare that the research was conducted in the absence of any commercial or financial relationships that could be construed as a potential conflict of interest.
